# Supplementary material for: Enhancing the expression of ARK1 genes in poplar leads to multiple branches and transcriptomic changes
Source: R Soc Open Sci. 2020 Sep 9;7(9):201201. doi: 10.1098/rsos.201201 (PMC7540752; doi:10.1098/rsos.201201)
Supplement: Supplementary Table1 [file rsos201201supp1.docx]

Supplementary Table1. Transcriptome sequencing quality of ‘717’ and ‘84K’ poplar.

| sample | library | | raw_reads | | clean_reads | | | clean_bases | error_rate | | Q20 | | Q30 | | GC_pct |
| --- | --- | --- | --- | --- | --- | --- | --- | --- | --- | --- | --- | --- | --- | --- | --- |
| ‘717’CK1 | FKDL171663363-1A | | 42,343,556 | | 41,627,978 | | | 6.24G | 0.02 | | 97.03 | | 92.56 | | 44.59 |
| ‘717’CK2 | FKDL171663364-1A | | 38,399,844 | | 37,780,938 | | | 5.67G | 0.02 | | 97.13 | | 92.74 | | 44.62 |
| ‘717’CK3 | FRAS190287287-1a | | 50,055,612 | | 48,467,618 | | | 7.27G | 0.02 | | 98.15 | | 94.4 | | 43.9 |
| ‘717’TR1 | FKDL171663365-1A | | 48,564,226 | | 47,794,780 | | | 7.17G | 0.02 | | 97.16 | | 92.8 | | 44.6 |
| ‘717’TR2 | FKDL171663366-1A | | 34,379,736 | | 34,001,932 | | | 5.1G | 0.02 | | 97.28 | | 93 | | 44.78 |
| ‘717’TR3 | FRAS190287288-1a | | 55,304,170 | | 54,017,012 | | | 8.1G | 0.02 | | 98.23 | | 94.52 | | 43.68 |
| ‘84K’CK_1 | | FRAS190118333-1a | | 40,477,334 | | 39,765,228 | 5.96G | | 0.03 | 97.9 | | 93.93 | | 44 | |
| ‘84K’CK_2 | | FRAS190118334-1a | | 40,137,628 | | 39,552,618 | 5.93G | | 0.03 | 97.62 | | 93.26 | | 44.15 | |
| ‘84K’CK_3 | | FRAS190118335-1a | | 46,083,018 | | 45,299,010 | 6.79G | | 0.03 | 97.64 | | 93.34 | | 44.07 | |
| ‘84K’T_1 | | FRAS190115115-1a | | 54,355,438 | | 53,528,906 | 8.03G | | 0.03 | 97.77 | | 93.57 | | 43.61 | |
| ‘84K’T_2 | | FRAS190115113-1a | | 49,067,640 | | 48,441,358 | 7.27G | | 0.03 | 97.68 | | 93.32 | | 43.69 | |
| ‘84K’T_3 | | FRAS190115114-1a | | 51,185,874 | | 50,514,268 | 7.58G | | 0.03 | 97.83 | | 93.71 | | 43.89 | |
